# Molecular Mechanisms and Treatment of Sarcopenia in Liver Disease: A Review of Current Knowledge

**DOI:** 10.3390/ijms22031425

**Published:** 2021-01-31

**Authors:** Hiroteru Kamimura, Takeki Sato, Kazuki Natsui, Takamasa Kobayashi, Tomoaki Yoshida, Kenya Kamimura, Atsunori Tsuchiya, Toshiko Murayama, Junji Yokoyama, Hirokazu Kawai, Masaaki Takamura, Shuji Terai

**Affiliations:** 1 Division of Gastroenterology and Hepatology, Graduate School of Medical and Dental Sciences, Niigata University, Niigata 951-8510, Japan; takeki.sato.pr.1.3@gmail.com (T.S.); kazukinatsui@gmail.com (K.N.); tkms0927@med.niigata-u.ac.jp (T.K.); tomomot1105@med.niigata-u.ac.jp (T.Y.); kenya-k@med.niigata-u.ac.jp (K.K.); atsunori@med.niigata-u.ac.jp (A.T.); yokoyaj@med.niigata-u.ac.jp (J.Y.); kawaih@med.niigata-u.ac.jp (H.K.); atmc@med.niigata-u.ac.jp (M.T.); terais@med.niigata-u.ac.jp (S.T.); 2 Nutrition Support Team, Niigata University Medical and Dental Hospital, Niigata 951-8510, Japan; tomura@unii.ac.jp

**Keywords:** sarcopenia, liver cirrhosis, hyperammonemia, BCAA, abnormal sex hormones

## Abstract

Sarcopenia is characterized by progressive and generalized loss of skeletal muscle mass and strength that occurs with aging or in association with various diseases. The condition is prevalent worldwide and occurs more frequently in patients with chronic diseases owing to the intrinsic relationship of muscles with glucose, lipid, and protein metabolism. Liver cirrhosis is characterized by the progression of necro-inflammatory liver diseases, which leads to fibrosis, portal hypertension, and a catabolic state, which causes loss of muscle tissue. Sarcopenia is of significant concern in the state of liver cirrhosis because sarcopenia has been associated with higher mortality, increased hospital admissions, worse post-liver transplant outcomes, decreased quality of life, and increased risk for other complications associated with cirrhosis. Therefore, sarcopenia is also an important feature of liver cirrhosis, representing a negative prognostic factor and influencing mortality. An increased understanding of sarcopenia could lead to the development of novel therapeutic approaches that could help improve the cognitive impairment of cirrhotic patients; therefore, we present a review of the mechanisms and diagnosis of sarcopenia in liver disease and existing therapeutic approaches.

## 1. Introduction

Sarcopenia was first defined by Rosenberg. The word originates from a combination of *sarx* and *penia*, which means “muscle” and “loss” in Greek, respectively, and has been defined as the “loss of skeletal muscle mass with aging” [1]. It has recently been registered as an injury/disease in the International Statistical Classification of Diseases and Related Health Problems, 11th revision [2].

Reports on the frequency of sarcopenia vary. In general, 1–29% of elderly people are affected, and a high proportion (14–33%) of people receiving long-term care have sarcopenia [3]. As no unified diagnostic criteria currently exist, the diagnosis is made at the discretion of the clinician. In early 2018, the European Working Group on Sarcopenia in Older People 2 (EWGSOP2) updated the original definition to reflect scientific and clinical evidence that has accumulated over the last decade. They reported that sarcopenia is indicated for cases with decreased skeletal muscle mass of the extremities in addition to decreased physical function (indicated by gait speed) or muscle strength (indicated by grip strength) [4].

Physical function declines with age as the balance between protein synthesis and degradation in the skeletal muscle becomes compromised. This reduces muscle strength, which can result in falls or gait disturbance [5]. A cohort study showed that muscle mass begins to decrease in both men and women in their late 40 s, with consequent negative influences on daily life [6].

**Figure 1:**
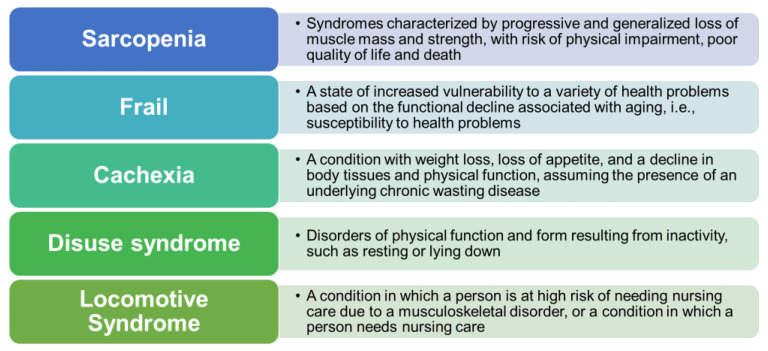
Sarcopenia and other related hypoactivity syndromes.

The various causes of decreased muscle activity include sarcopenia, flail, cachexia, disuse syndrome, and locomotive syndrome (Figure 1). These syndromes have partly overlapping phenotypes.

Figure 1 shows definitions with some overlap, each involving a decline in physical function [7].

In this study, we review the mechanisms and diagnosis of sarcopenia in liver disease and the existing and potential novel therapeutic approaches.

## 2. Classification of Sarcopenia

Recent classification systems largely categorize sarcopenia into primary and secondary sarcopenia (Table 1). Losses of muscle mass and strength with aging are classified as primary sarcopenia, whereas losses of muscle mass and strength due to disuse muscle atrophy related to activity, disease, and nutrition status are classified as secondary sarcopenia [8]. Secondary sarcopenia usually involves some underlying disease, such as renal, liver, inflammatory disease, or malignant tumor, with a concurrent lack of protein and energy intake. Sarcopenia that develops from liver disease is an example of secondary sarcopenia. The Sarcopenia Judgment Criteria (Version 1) for liver diseases developed by the Japan Society of Hepatology Sarcopenia Judgement Criteria Creating Working Group refer to the diagnostic criteria and definition of primary sarcopenia proposed by the Asian Working Group for Sarcopenia (AWGS) [9].

## 3. Mechanism of Sarcopenia in Liver Cirrhosis

Liver cirrhosis with a background of viral hepatitis frequently leads to sarcopenia, and its incidence has been reported to be 30% in patients with chronic hepatitis and 40% in patients with liver cirrhosis [10]. Thus, sarcopenia should be considered because it can affect the quality of life of patients with cirrhosis. A recent study involving computed tomography (CT) analysis of skeletal muscle mass index (SMI), which was performed as part of an interventional radiology treatment for hepatocellular carcinoma, reported that high levels of skeletal muscle mass loss during six months of treatment were associated with poor prognosis [11]. Skeletal muscle mass decreases progressively with age, at an estimated rate of approximately 1% per year, as aging puts the body in a state where the synthesis of the skeletal muscle is exceeded by its degradation. This decrease occurs at higher rates in people with low levels of activities of daily living (ADL) [12].

Sarcopenia in liver cirrhosis is multifactorial. Changes due to liver cirrhosis are considered potential disruptors of the balance of protein anabolism and catabolism. Muscle mass is regulated by the balance of protein anabolism and catabolism and decreased muscle mass due to decreased anabolism and enhanced catabolism is a characteristic of sarcopenia.

In addition to decreased protein anabolism as a result of decreased hepatic function, the factors that induce sarcopenia in liver disease include hyperammonemia, lower levels of branched-chain amino acids (BCAAs), and abnormal sex hormone levels (lower testosterone levels) [13]. These are discussed in detail below.

**Figure 2:**
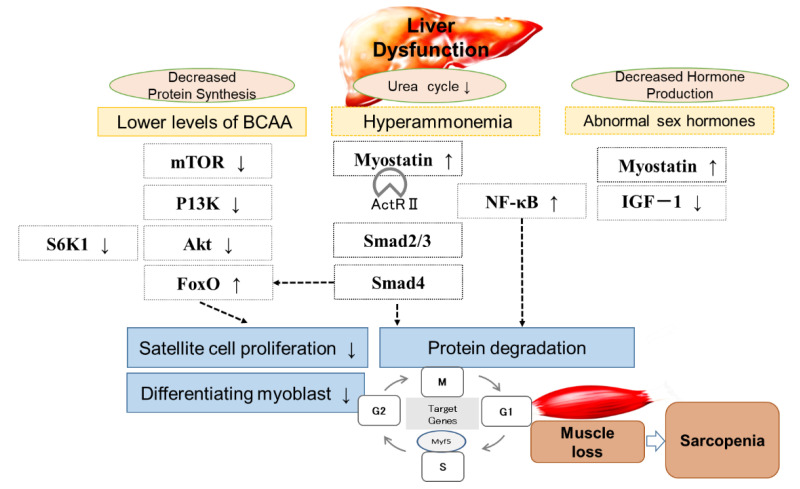
Mechanism of liver disease and sarcopenia.

Figure 2 shows a schematic overview of the crosstalk between liver cirrhosis and the mechanisms of muscle loss and sarcopenia, lower BCAA levels, hyperammonemia, and abnormal sex hormone levels. The expression patterns during the different phases of the cell cycle are also shown in Figure 2. The myogenic factor 5(Myf5) protein level peaks in G0 (Gap0) phase, decreases during G1 (Gap1) phase, and then increases again at the end of G1, at which they remain stable through mitosis signaling activated by insulin-like growth factor 1 (IGF1), positively regulating muscle mass, primarily via the induction of protein synthesis downstream of serine/threonine-protein kinases (Akt) and mammalian target of rapamycin (mTOR) [14] myostatin/GDF11/activin pathway negatively regulates muscle size as a result of the phosphorylation of SMAD2/3 primarily by inhibiting Akt. IGF1 acts via the IGF receptor (IGFR) and insulin receptor substrate 1 (IRS1) activates Akt. Akt activates mTOR complex 1 (mTORC1), a multiprotein complex that requires the protein raptor for its function and is acutely inhibited by FKBP/rapamycin. mTORC1 controls protein synthesis by phosphorylating S6 kinase 1 (S6K) [15].

### 3.1. Hyperammonemia

The risk of hyperammonemia increases with the increase in loss of muscle mass along with liver dysfunction, as the muscle helps in NH_3_ disposal. Ammonia is produced in the gastrointestinal tract by the bacterial degradation of amines, amino acids, purines, and urea. Enterocytes also converts glutamine to glutamate and ammonia through the activity of glutaminase. Normally, ammonia is detoxified in the liver by conversion to urea in the urea cycle [16]. Ammonia is also consumed in the conversion of glutamate to glutamine, a reaction that depends on the activity of glutamine synthetase. Two factors contribute to hyperammonemia, which occurs in patients with cirrhosis. First, the mass of functioning hepatocytes decreases, resulting in fewer opportunities for ammonia to be detoxified through the above-mentioned processes. Second, portosystemic shunting (e.g., spleno-renal and gastro-renal shunts) divert ammonia-containing blood from the liver to the systemic circulation. Normal skeletal muscle cells do not possess the enzymatic machinery required for the urea cycle but do contain glutamine synthetase. Glutamine synthetase activity in muscles increases in cases of cirrhosis and portosystemic shunting; thus, the skeletal muscle is an important site for ammonia metabolism in cirrhosis [17].

Myokines are cytokines synthesized and released by myocytes. Myostatin is a major myokine that induces a negative feedback mechanism in muscles. The effects of myostatin are mediated through the activin type IIB receptor, which is expressed ubiquitously in the skeletal muscle [18,19]. The downstream mediators of myostatin, SMAD2 and SMAD3, are phosphorylated and form a complex with SMAD. This complex, in turn, stimulates forkhead-box-containing protein O (FoxO)-dependent transcription and regulates the transcription of genes associated with proliferation and differentiation in skeletal muscle precursor cells and the protein degradation pathways of ubiquitin-proteasome processes and autophagy in mature myofibers [20,21]. Besides, myostatin-mediated SMAD signaling activation inhibits protein synthesis in muscle tissues by suppressing the signaling pathway of the Akt-mediated mammalian target of rapamycin (mTOR). Functionally, myostatin is a negative regulator of muscle growth, thereby leading to the inhibition of myogenesis through muscle cell differentiation and growth [22]. In addition, hyperammonemia leads to myostatin expression via a nuclear factor of the kappa light polypeptide gene enhancer in the B-cell (NF-κB)-dependent toll-like receptor-independent pathway [20].

Myostatin, another Transforming Growth Factor-β (TGFβ) family member with established cachectic and profibrotic properties [23], is produced by muscles and inhibits muscle growth by acting through either activin-like kinase 4 (Alk 4) or activin-like kinase 5 (Alk 5) and the activin type II receptors [24] This indicates that myostatin shares a signaling pathway with the activins and that follistatin also binds, albeit less effectively, to myostatin. The ability of follistatin to prevent muscle wasting in cachexia and fibrosis in mouse models of muscular dystrophy probably involves interference with myostatin signaling via the pathway, although the relative contributions of endogenous activin and myostatin have not been well characterized in these models [25,26,27].

### 3.2. Lower Levels of BCAAs

Valine, leucine, and isoleucine are essential amino acids for humans and are involved in the pathophysiology of liver diseases. The high protein requirements in cirrhosis of the liver can be explained because of an accelerated rate of transition from a fed state to a fasted state, possibly due to diminished liver glycogen stores. When glycogen stores are insufficient to ensure normal blood glucose levels even during short-term fasting, the early onset of gluconeogenesis from amino acids will occur as a result. This leads to additional amino acid loss and depletion of tissue protein stores [28].

BCAAs are involved in the metabolism of proteins, glucose, and fat. BCAAs activate mTOR signaling, stimulating the synthesis of glycogen and proteins such as albumin. mTOR is an atypical protein kinase that controls growth and metabolism in response to nutrients, growth factors, and cellular energy levels, and it is frequently dysregulated in liver cirrhosis [29]. BCAAs regulate the metabolism of glucose and lipids through the PI3K-Akt pathway. Deficiencies in BCAAs reduce hepatic fatty acid synthesis, promote fatty acid β-oxidation, and increase fat mobilization in white adipose tissue through the AMP-activated protein kinase-mTOR-FoxO1 pathway.

A highly conserved signaling pathway involving insulin-like growth factor 1 (IGF1) and a cascade of intracellular components are related to the regulation of skeletal muscle growth. The P13K protein kinase B (PKB)/Akt-mTOR pathway, a central component in this cascade in protein anabolism, exists downstream of the IGF1 signaling pathway [30]. The pathway affects mTOR, glycogen synthase kinase 3β, and protein degradation via the transcription factors of the FoxO family.

Phosphorylation of ribosomal protein S6 kinase 1 by mTOR initiates protein synthesis via activation of the S6 ribosomal protein and other translation-related proteins. Growth hormones that act on the liver stimulate the secretion of IGF; therefore, protein anabolism decreases with age owing to the decline in growth hormone levels and hepatic function that occur with aging [31].

Insulin resistance causes a similar decrease in protein anabolism [32]. Protein catabolism is brought about by the ubiquitin-proteasome and autophagy systems, which are positively controlled by the transcription factor FoxO [33]. The effects of FoxO are suppressed by the phosphorylation from the PKB/Akt complex and subsequent transportation out of the nucleus. Autophagy is the process by which autophagosomes form and fuse with lysosomes to degrade cytoplasmic components such as proteins, which are positively controlled by FoxO and suppressed by mTOR [34].

Protein-energy malnutrition (PEM) is defined as a state in which protein and energy intake are insufficient. In the context of liver disease, this involves an imbalance between plasma levels of free amino acids, and hypoproteinemia and hyperammonemia, as well as a negative nitrogen equilibrium resulting from abnormal protein/amino acid metabolism due to decreased efficiency of glucose use during the early morning fasting state [35]. This state is equivalent to three days of fasting by a healthy person. During fasting, liver glycogen reserves decrease, and gluconeogenesis occurs, using amino acids from the breakdown of myoproteins; therefore, the skeletal muscle mass decreases, and the nitrogen balance tends to become negative. In the postabsorptive state, body fat is used as a nutrient supply. Among patients with liver cirrhosis, 43% were in a state of energy malnutrition, 61% were in a state of protein malnutrition, and 27% were in a state of PEM [36].

The three BCAAs (valine, leucine, and isoleucine) play important roles in the formation and maintenance of the skeletal muscle [37]. Decreases in their levels in the context of liver disease can lead to decreased muscle mass regardless of age [38]. Furthermore, decreased BCAA levels in patients with liver cirrhosis can result in amino acid imbalances and a decrease in the Fischer ratio [39]. One plausible mechanism of amino acid imbalance is that the decline in the detoxification function of the liver that occurs during cirrhosis is compensated by the inhibition of ammonia metabolism in the skeletal muscles, and BCAAs are used as substrates in this process [40].

### 3.3. Abnormal Sex Hormones

The liver plays an essential role in energy homeostasis and is involved in hormone metabolism. The reproductive functions of the testes and ovaries decline with age [41]. The common clinical features of cirrhosis include feminization of body shape and gynecomastia, which are primarily due to a decrease in testosterone levels and a concomitant increase in the estrogen-to-androgen ratio. This change affects muscle protein turnover, which leads to the suppression of myoblast differentiation to skeletal muscle cells, resulting in sarcopenia. Testosterone specifically targets androgen receptors in existing muscle cells to promote growth and in satellite cells to trigger differentiation into new myocytes; however, it may also act on other pathways, including the downregulation of myostatin and upregulation of IGF-1, which may contribute to its efficacy in cirrhosis [42]. Multiple factors most likely contribute to testosterone deficiency in cirrhosis, and all levels of the hypothalamic-pituitary-testicular axis can be affected. Some cases of alcoholic cirrhosis result in a direct testicular injury caused by ethanol and elevated luteinizing hormone (LH) levels, consistent with primary hypogonadism [43]. More commonly, cirrhosis is associated with central hypogonadism, where pituitary LH production is either inappropriately normal or even suppressed, despite low circulating testosterone levels. In end-stage cirrhosis, LH is almost universally low.

A severe systemic disease of any etiology, including liver failure, can downregulate gonadotropin-releasing hormone secretion by the hypothalamus and lead to secondary testicular failure. This is believed to be at least partly due to the direct effects of elevated inflammatory cytokines such as IL-1, IL-6, and tumor necrosis factor-alpha [44].

Given the strong association between sarcopenia and the risk of mortality in patients with cirrhosis, therapies that increase muscle mass are important for decompensated cirrhosis. The ability of testosterone therapy to increase muscle mass in this setting is notable given the multiple factors that contribute to sarcopenia in cirrhosis. These factors include malnutrition, portal hypertension, elevated inflammatory mediators, reduced IGF-1 level, myostatin upregulation, and a shift to muscle protein breakdown for energy use as a result of reduced hepatic glycogen synthesis and storage [45].

## 4. Modalities for the Assessment of Sarcopenia in the Clinical Field

The Japan Society of Hepatology (JSH) has evaluated grip strength and total muscle mass at the third lumbar vertebra (L3) by using computed tomography (CT) and bioelectrical impedance analysis. This evaluation confirmed that when the Sarcopenia Judgment Criteria (Version 1) for liver diseases of the JSH [8] are fulfilled, the diagnosis of sarcopenia is appropriate (Table 2).

Given the high mortality rate associated with decreased gait speed, this parameter is considered important in the evaluation of ADL. Gait speed evaluation, according to the AWGS, is performed along a distance of ≥6 m to assess normal gait speed without acceleration or deceleration. The patient was asked to walk from 0 to 6 m, and the time taken for the patient to walk 4 m between the 1 and 5 m markers was measured. Decreased gait speed is defined as ≤0.8 m/s (a gait speed of 1 m/s is the speed that is sufficient for a person to cross a pedestrian-crossing at a safe speed) [9]; however, measuring gait speed as part of routine medical practice in outpatient visits is challenging because only 3.6% of elderly patients receiving home-based care in Japan fulfill the definition of decreased gait speed. Version 1 of the JSH guidelines does not include gait speed as an indicator of sarcopenia.

Grip strength is a simple and reliable indicator of muscle strength and is included in the JSH Sarcopenia Judgment Criteria [8]. The value correlated with lower limb muscle strength (low grip strength is a clinical indicator of low mobility) and grip strength is a better predictor of clinical outcome than muscle mass [45].

A Smedley Grip Tester is commonly used to assess grip strength. The patient holds the grip meter outward in an upright position and opens and closes the hand while keeping the arm raised. Two measurements were taken for each hand, and the average value was assumed to be the grip strength value. The cutoff value for low grip strength according to the JSH guidelines is <26 kg for men and <18 kg for women [8].

Other than the modalities recommended in the JSH, the judgment criteria such as those of the EWGSOP and AWGS recommend dual-energy X-ray absorptiometry (DXA) for the evaluation of sarcopenia [9]. This method is used to measure bone mineral content to diagnose osteoporosis and determine therapeutic effects. It also enables the measurement of body fat mass and fat-free mass. Based on the principle that the attenuation of the energy of X-rays passing through a substance depends on the nature and amount of the substance, the bone mineral content, body fat mass, and fat-free mass can be measured from the difference in the transmittance of the two types of X-rays with different energies [46].

The use of X-ray methods has several advantages, including minimal radiation exposure, little effect on the human body, and its non-invasive nature. Although nonfat mass measurement by DXA does not indicate pure muscle mass, in the case of the limbs, most of the mass that is not represented by fat or bone can be attributed to the muscle. The SMI is obtained by dividing the total nonfat mass in the limbs by the square of height.

The principle of bioelectrical impedance analysis (BIA) is based on the determination of body composition based on the difference in impedance (electrical resistance) relative to the alternating current. Impedance is high in fat tissue and bones (where the water content is low) and low in lean tissues because of their high water content. No radiation exposure is involved, and the procedure is short and minimally invasive. As most lean tissue is muscle, it is possible to estimate muscle mass using this approach; however, care must be taken when interpreting the results of BIA in cases in which large amounts of ascites or significant edema are present because excess water is stored in the muscle tissue. [47].

CT can provide important quantitative data on the composition of muscles and the distribution of adipose tissue. This modality is often used in examinations for hepatocellular carcinoma in patients with liver cirrhosis, even in those with decompensated cirrhosis who have abdominal ascites. The measurement of muscle mass with CT generally measures the difference in Hounsfield units (HU), which is the signal intensity of X-ray CT between tissues. CT images are defined by relative readings of absorption, with water being 0 HU and air being −1000 HU. Fats are defined by readings between −30 and −190 HU, and areas measured to be 0–100 HU represent muscles. The muscle area at the level of the third lumbar vertebra (L3) is indicative of the total muscle mass and is often imaged in the case of liver disease. The standard and temporally defined normal levels (men: 42 cm^2^/m^2^, women: 38 cm^2^/m^2^) were defined using the Slice-O-Matic software (Tomovision, Montreal, Canada). In facilities that do not have this muscle-measuring software, acceptable methods of diagnosing sarcopenia are temporal, which is defined as the left and right total of the long axis multiplied by the short axis of the iliopsoas muscle at the L3 level (cutoff value: 6.0 cm^2^/m^2^ for men and 3.4 cm^2^/m^2^ for women) or the psoas muscle index measured using the manual trace method (cutoff value: 6.36 cm^2^/m^2^ for men and 3.92 cm^2^/m^2^ for women) [48].

To date, some serological indicators for sarcopenia have been reported but they are not disease-specific marker. Based on the results of highly sensitive analyses, biochemical markers, including C-reactive protein, immune complex C1q, C-terminal agrin fragment, telomere length, and p53 codon 72 gene polymorphisms, have been reported as substitutes for sarcopenia [49]. In addition, serum protein levels reflect the visceral protein content and are important indicators of protein malnutrition [50].

In contrast, levels of rapid-turnover proteins such as prealbumin, transferrin, and retinol-binding protein are suitable as dynamic nutritional indexes for the evaluation of short-term nutritional status [51].

## 5. Other Effects of Sarcopenia Related to Liver Cirrhosis

Sarcopenia is also related to prescribed medicine for liver cirrhosis and complications in other organs (Table 3). In general, restriction of protein intake is required to maintain appropriate renal function during the chronic renal failure phase. Renal dysfunction is a common manifestation of advanced cirrhosis that is associated with significant mortality and morbidity. Liver cirrhosis patients with chronic renal failure are at risk of developing complications associated with nutritional disorders [52]; therefore, protein restriction also causes secondary sarcopenia. Considering this protein restriction, target intake levels must be decided according to each patient’s disease, age, physical activity level, complications, and ADL, rather than using a uniform approach. Diet therapy has also been suggested to be prioritized for patients aged >75 years [53].

In 2017, the Na-K-Cl co-transporter, which is the target of loop diuretics, was reported to regulate myoblast differentiation and skeletal muscle hypertrophy. Administration of loop diuretics suppresses skeletal muscle differentiation and hypertrophy [54]. Although loop diuretics are believed to be involved in sarcopenia in this manner, a recent report has suggested similar clinical results in patients with liver cirrhosis [55].

The pathology of sarcopenic obesity has attracted attention in recent years; it occurs when chronic inflammation in obesity causes insulin resistance and oxidative stress. These factors lead to a quantitative and qualitative decline in muscle mass and an increase in fat, which together cause sarcopenia [56]. Obesity is a common complication of type 2 diabetes, and epidemiological studies have shown enhanced decreases in muscle strength and mass of the lower extremities in elderly patients with these conditions [57,58]. Current evidence demonstrating the effect of obesity on muscle quality is limited [59].

High-fat diets cause changes to the normal intestinal bacteria, which can result in inflammation, and the involvement of intestinal bacteria in the underlying pathologies of obesity, type 2 diabetes, and metabolic syndrome has been demonstrated [60].

Inflammation that arises from the disturbance of intestinal flora may be involved in mitochondrial dysfunction that occurs with aging, which in turn leads to the release of mitochondrial damage-associated molecular patterns such as mitochondrial deoxyribonucleic acid and adenosine triphosphate. These molecules represent novel therapeutic targets for sarcopenia, which have been reported to be involved in muscle loss [61].

## 6. Nutritional Status in Liver Cirrhosis

Objective evaluations of the extent, characteristics, and sustained period of nutritional abnormalities in liver disease are essential for proper nutritional management. A nutritional assessment can be functionally classified as either static or dynamic.

A static assessment evaluates slow metabolic turnover using measurements of body composition, including triceps skinfold thickness and arm muscle circumference (AMC) [62] as well as immunological indicators such as peripheral blood lymphocyte count, delayed skin hypersensitivity, and lymphocyte blast transformation. These measurements were used to assess nutritional status.

A dynamic assessment analyzes the fluctuation of metabolism during the measurement period and is used to judge the short-term effects of nutritional treatments. For example, protein metabolism is assessed using the respiratory quotient based on indirect calorimetry [63].

Many patients with liver cirrhosis have decreased energy intake levels because of taste disorders and changes in gastrointestinal hormones, inflammatory cytokines, and ascites levels. Measurement of energy metabolism by indirect calorimetry can be used to examine the enhancement of resting energy expenditure in patients with liver cirrhosis. Furthermore, the combustion ratio of energy substrates can be used to evaluate glucose and fat combustion in the case of liver disease [64].

Although no gold standard method has been established for nutritional assessments in liver disease, assessments are generally performed by evaluating nutritional intake and body composition as well as with serological assessments, including subjective global assessment, which is based on medical history, medical interviews, and physical findings. This method is simple and applicable to patients with liver cirrhosis [65]. The nonprotein respiratory quotient (npRQ) is recommended for the assessment of energy malnutrition; however, given its complexity, it is rarely used in routine medical practice. The %AMC and free fatty acid (FFA) levels in the early morning fasting state correlated with the npRQ, where %AMC < 95 and FFA > 660 μEq/L indicate npRQ < 0.85 [66]. In liver cirrhosis, glycogen storage is drastically reduced owing to abnormalities in glycogen synthesis. The patient starves easily even after a short fasting period, and the body rapidly enters a state of malnutrition through the breakdown of proteins and degradation of body fat, as patients with liver cirrhosis tend to undergo intense starvation, which is equivalent to 2–3 days of fasting in healthy people at the time of waking [67].

Version 3 of the Japanese Society for Parenteral and Enteral Nutrition: Intravenous Enteral Nutrition Guidelines states that consuming a late evening snack is effective in avoiding a starvation state from decreased glycogen storage during the night and that in addition to a general diet and intake of supplements, nutritional status can be improved by ingesting BCAA granule preparations and enteral nutrients for liver failure before going to bed [68]. As protein synthesis in the liver primarily occurs at night and the rate-limiting step of protein synthesis in patients with liver cirrhosis is the lack of BCAA, it is rational to take enteral nutrition containing BCAAs as part of the treatment for liver failure [69]. Late evening snacking (LES) decreased lipid oxidation and improved nitrogen balance, irrespective of the composition or type of formulation used. Daytime isocaloric isonitrogenous snacks did not have the metabolic or clinical benefit of LES. LES decreased skeletal muscle proteolysis. LES holds the most promise as an intervention to reverse anabolic resistance and sarcopenia with improved quality of life in patients with cirrhosis. Its long-term benefit and improved survival require critical evaluation [70].

## 7. Treatment of Sarcopenia in Liver Cirrhosis

**Figure 3:**
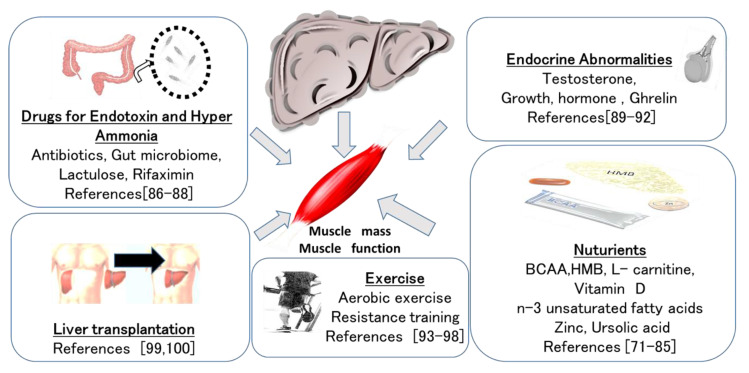
Treatment interventions for liver disease and sarcopenia.

The effects of each treatment on decreased muscle mass are summarized in Figure 3.

### 7.1. Drug Medicine and Nutrients

Protein synthesis within muscle cells is necessary to maintain muscle mass. Changes in the concentrations of blood amino acids can modulate skeletal muscle protein metabolism, and increased concentrations from amino acid intake will enhance protein synthesis. A prospective study reported that low protein intake leads to a decrease in skeletal muscle mass [71].

A significant increase in lean body mass was suggested to increase the rate of protein synthesis in muscles [72]. Valine, leucine, and isoleucine are BCAAs that are present in approximately 30–40% of muscle proteins and are involved in the synthesis of muscle proteins and suppression of protein breakdown. Leucine is known to stimulate muscle protein synthesis by activating the mTOR pathway [73]. A previous study showed that supplementation with BCAAs activates cell signaling pathways that result in increased myofibrillar protein synthesis. Although the administration of BCAAs alone may not induce a maximal myofibrillar protein synthesis response to resistance exercise training, supplementation with BCAAs may be a useful approach for increasing muscle mass in patients with liver cirrhosis [74].

Recently, β-hydroxy-β-methylbutyrate (HMB) has attracted attention because of its effect on muscle synthesis, which is believed to be more potent than that of leucine. Administration of HMB and supplements containing HMB has been shown to increase muscle mass and strength in various clinical conditions, although the effect size was small [75]. HMB is a bioactive metabolite formed from the breakdown of the branched-chain amino acid leucine. HMB is responsible for the enhancement of protein anabolism and suppression of catabolism in muscles [76].

Carnitine is required for the transportation of fatty acids from the cytoplasm into the mitochondria. In humans, carnitine is not only absorbed through food but is also synthesized in the kidneys, liver, and brain. L-carnitine is involved in the functional activation of mitochondria, and a study that compared a group treated with carnitine and a control group that had lipopolysaccharide-induced inflammation revealed correlations between carnitine and muscle mass, the muscle-specific protein MuRF1, mitochondrial function, and myokine [77].

Several studies have shown correlations between muscle mass and muscle strength and blood vitamin D levels, and their findings suggest that treatment with vitamin D compounds results in sustained increases in vitamin D receptor gene expression in human skeletal muscle, suggesting that vitamin D exerts a direct action on muscles in addition to its influence on bone and muscle metabolism [78].

The relationship between n-3 unsaturated fatty acids and skeletal muscle is a subject of growing interest. Metabolites of n-3 long-chain unsaturated fatty acids such as eicosapentaenoic acid (EPA; 20:5 n-3) and docosahexaenoic acid (22:6 n-3) have been reported to promote β-oxidation of fatty acids in the skeletal muscle and suppress inflammation and muscle loss [79]. While multiple reports have claimed that EPA is effective for the prevention of skeletal muscle mass loss due to cancer cachexia. Administration of n-3 fatty acids has been shown to increase the rate of protein synthesis in people aged ≥65 years [80]; therefore, supplementation of n-3 fatty acids, in addition to amino acids, may help increase protein synthesis in muscles as part of sarcopenia treatment [81].

Minerals may contribute to the prevention and treatment of sarcopenia, age-related loss of muscle mass, muscle strength, and physical performance. Minerals may be important nutrients to prevent and treat sarcopenia. Zinc deficiency can also lead to a nitrogen metabolic disorder in patients with liver cirrhosis. Zinc supplementation can improve not only ammonia metabolism but also protein metabolism. Zinc is an important trace element for normal cell development, proliferation, and differentiation, and is also known to be crucial for ensuring an appropriate immunological reaction, such as anti-inflammatory effects, anti-oxidant effects, or autophagy [82].

Zn deficiency can cause a wide spectrum of clinical presentations, including appetite loss, body hair loss, impaired taste and smell, testicular atrophy, cerebral and immune dysfunctions, and impairment of drug excretion ability, which are frequently observed in chronic liver diseases (CLDs) because Zn homeostasis is primarily regulated in the liver. Albumin synthesis disability can cause Zn deficiency in patients with liver cirrhosis. Serum Zn concentrations inversely correlated with serum ammonia levels in LC patients [83].

Ursolic acid (UA), a natural pentacyclic triterpenoid widely distributed in medicinal herbs and fruits, possesses a wide range of biological activities [84].

In addition to increased skeletal muscle Akt activity, UA has been shown to induce skeletal muscle hypertrophy and increase exercise capacity in a mouse model of diet-induced obesity. These results indicate that UA supports resistance-exercise-induced mTORC1 activity and improves sarcopenia [85].

In a sarcopenia rat model produced using a portosystemic shunt, oral administration of ornithine, aspartic acid, and rifaximin for four weeks was shown to increase skeletal muscle mass. Moreover, the same treatment was reported to increase type 2 myofibers (which readily consume carbohydrates), suppress the expressions of myostatin and autophagy, and activate mTOR [86]. Lactulose and rifaximin, which are administered to treat hepatic encephalopathy, lower blood ammonia levels [87]. As ammonia consumes BCAAs when it is detoxified in the muscle and accelerates myostatin production and autophagy, ammonia-lowering drugs may improve sarcopenia [88].

Steroids have been used in studies of male hormone androgens. A double-blind study of the use of steroids in male patients aged ≥65 years demonstrated decreased fat mass and improved physical function in response to testosterone adhesive agents (selective androgen receptor modulators) [89]. Testosterone has been indicated as a risk factor for the development of liver cancer, however, large-scale clinical studies are needed to evaluate the influence of steroids on liver carcinogenesis and prognosis [90].

In addition to the potential mortality benefit of attenuating sarcopenia, a previous study showed that the nonmuscle effects of testosterone therapy could also positively influence long-term outcomes. A significant increase in hemoglobin was observed in an anemic cohort without causing any polycythemia, and in other populations, anemia was associated with increased all-cause mortality. The study also showed that testosterone therapy reduced the level of hemoglobin A1c (a surrogate marker of insulin resistance), which is in keeping with trials in other hypogonadal populations, demonstrating reduced insulin resistance with testosterone therapy [91].

Ghrelin, which comprises a peptide of 28 amino acid residues, is produced primarily in the stomach. It controls food intake through the activation of the hypothalamic nuclei one of the most potent and long-lasting appetite stimulators. Although the direct effect of ghrelin on skeletal muscle remains unknown, increasing the administered amount of ghrelin has been reported to improve nutritional status, increase muscle strength, suppress sympathetic nerve activity, and manifest anti-inflammatory effects [92].

### 7.2. Appropriate Exercise for Patients with Liver Disease

The most qualified advice about exercise for a patient with chronic disease is to “start low, progress slowly, and be alert for symptoms”. Exercise is differentiated from physical activity as it is planned and performed on a repeated basis over an extended period of time for the purpose of improving fitness, performance, and health [93].

The exercise training prescription provided to each patient follows the FITT principles: Frequency, Intensity, Time, Type of exercise.

After exercise, BCAAs help to increase muscle mass and improve insulin resistance and glucose uptake in the muscles [94].

Nutritional supplementation alone is inadequate for increasing skeletal muscle mass; however, combining supplementation with exercise three times a week (20–30 min per session), consisting of aerobic exercise and resistance training has been reported to be effective [95].

Although endurance training does not promote muscular hypertrophy, resistance training has been shown to produce significant hypertrophy in slow muscles [96]. The intended outcomes of resistance exercise and nutritional supplementations are muscle strengthening, improvement of muscle quality, increased rate of myofibrillar metabolism, muscle fiber enlargement, and stimulation of muscle anabolism and anti-catabolism effects. An example of an effective exercise regimen could be as follows: two to three sets of 8–12 repetitions involving lifting a weight that is 80% of the maximum weight that can be lifted, performed three times a week for ≥3 months continuously [97].

A large amount of evidence exists showing that both low-load training (≤60% of one repetition maximum (1RM)) and high-load training (>60% of 1RM) are effective, whereas no significant differences have been found for isometric strength between the conditions. Changes in the measures of muscle hypertrophy were similar between the conditions. This is particularly important for the perception of fatigue and effort in older adults [98].

### 7.3. Fluctuations after Liver Transplantation

Although sarcopenia is highly likely to improve after liver transplantation [99], immunosuppressants (including steroids and calcineurin inhibitors), which are commonly used after surgery, can cause fluctuations in sarcopenia indicators such as muscle mass [100]. Although clinical improvement is not directly proportional to muscle function and turnover, sarcopenia worsens the prognosis in terms of ADL. The mechanisms involved are still unclear.

## 8. Conclusions

Sarcopenia is related to a poor prognosis and the deterioration of ADL. In our study, we reviewed the mechanism and diagnosis of sarcopenia in liver disease and the existing and potential novel therapeutic approaches. This review could serve as a useful reference to help improve the prognosis of the disease and ameliorate the decline in patients’ ADL.

## Figures and Tables

**Table 1:** Classification of sarcopenia.

Classification of Sarcopenia	Causes
**Primary sarcopenia**	
Age-related	No causes other than aging
**Secondary sarcopenia**	
Activity-related	A bedridden state or weightlessness
Disease-related	Severe organ (heart, lung, liver, kidney, or brain) dysfunction, inflammatory disease, or malignant tumor
Nutrition-related	Energy and protein deficiencies associated with malabsorption, gastrointestinal disease, and/or medication

**Table 2:** Parameters and criteria for the diagnosis of sarcopenia.

Parameter	Criteria for Diagnosing Sarcopenia
Muscle mass at L3 measured with CT	Men: 42 cm^2^/m^2^
	Women: 38 cm^2^/m^2^
SMI BIA	Men: 7.0 kg/m^2^
	Women: 5.7 kg/m^2^
Grip strength	Men: <26 kg
	Women: <18 kg

Abbreviations: BIA, bioelectrical impedance analysis; CT, computed tomography; L3, level of the third lumbar vertebra; SMI, skeletal muscle mass index.

**Table 3:** Other Effects of Sarcopenia Associated with Liver Cirrhosis.

Factor	The Key Mechanism	References
Renal Dysfunction-associated Liver Cirrhosis	Restriction of Protein Intake	[52,53]
Loop Diuretic Sarcopenia	Regulation for Myoblast Differentiation	[54,55]
Sarcopenic Obesity	Qualitative Decline in Muscle Mass for Insulin Resistance	[56,57,58,59]
Enterobacteria	Mitochondrial Dysfunction for Disturbance of Intestinal Flora	[60,61]
